# Membrane charge and lipid packing determine polymyxin-induced membrane damage

**DOI:** 10.1038/s42003-019-0297-6

**Published:** 2019-02-18

**Authors:** Adree Khondker, Alexander K. Dhaliwal, Sokunthearath Saem, Ahmad Mahmood, Cécile Fradin, Jose Moran-Mirabal, Maikel C. Rheinstädter

**Affiliations:** 1 Department of Physics and Astronomy, McMaster University, Hamilton, ON Canada; 2 Origins Institute, McMaster University, Hamilton, ON Canada; 3 Department of Chemistry and Chemical Biology, McMaster University, Hamilton, ON Canada; 4 Department of Biochemistry and Biomedical Sciences, McMaster University, Hamilton, ON Canada

## Abstract

With the advent of polymyxin B (PmB) resistance in bacteria, the mechanisms for *mcr*-1 resistance are of crucial importance in the design of novel therapeutics. The *mcr*-1 phenotype is known to decrease membrane charge and increase membrane packing by modification of the bacterial outer membrane. We used X-ray diffraction, Molecular Dynamics simulations, electrochemistry, and leakage assays to determine the location of PmB in different membranes and assess membrane damage. By varying membrane charge and lipid tail packing independently, we show that increasing membrane surface charge promotes penetration of PmB and membrane damage, whereas increasing lipid packing decreases penetration and damage. The penetration of the PmB molecules is well described by a phenomenological model that relates an attractive electrostatic and a repulsive force opposing insertion due to increased membrane packing. The model applies well to several gram-negative bacterial strains and may be used to predict resistance strength.

## Abstract (web summary)

Adree Khondker et al. identify new mechanisms by which the membrane surface and lipid packing in the bacterial membrane control the penetration of antibiotics such as of polymyxin B. These findings indicate that biophysical factors might influence the resistance strength of bacteria to antibiotics.

## Introduction

With the global emergence of antibiotic resistant bacteria, there is a need for new antimicrobial candidates^1,2^. Lipopeptides, amphiphillic peptides attached to a lipid, often show strong bactericidal efficacy through membrane rupturing^3^. Polymyxin B (PmB) is a model lipopeptide from which many analogous antibiotics have been developed, and various models such as the carpet, barrel-stave, and toroidal pore have shed insights onto the mechanisms of membrane rupturing^4^. Membrane charge and stability seem to play crucial roles in the molecular mechanism, efficacy, and synergy of these lipopeptides^3,5,6^.

Polymyxin resistance has recently been reported from the emergence of *mcr*-1 in bacteria, a transferrable gene encoding for a phosphatidylethanolamine transferase enzyme, which mediates the addition of an ethanolamine to lipid A on the bacterial membrane^7–9^. This alteration often reduces the membrane charge gradient and increases lipid packing. Together, these changes have been shown to reduce the bacteriocidal activity by lipopeptide antibiotics^10–13^.

PmB consists of a cyclic head group composed of di-aminobutyric acid residues adjoined to a hydrophobic acyl tail^14^. Molecular Dynamics simulations have shown the molecule to adhere to the surface of a membrane and form aggregates prior to penetrating the bilayer^15^. The di-aminobutyric acid residues anchor it to the bacterial membrane, and it has been found that the polypeptide ring is responsible for causing membrane permeability and damage^16–18^. One proposed mechanism is that PmB forms self-aggregates that can cause “pinching” of the inner- and outer-bacterial membranes, leading to subsequent membrane collapse^19^. However, there is still much debate upon a precise mode of action subsequent to peptide anchoring. The initial insertion of a single PmB is believed to be the crucial step in the formation of a membrane pore. Although the molecular details and determinants of this membrane penetration still need to be clarified, is it evident that membrane adherence and rigidity play a major role in lipopeptide pore formation^4,20,21^.

We prepared membrane assays that enabled us to investigate the effects of membrane charge and lipid packing on PmB attraction and insertion. We studied model membranes of 1-palmitoyl-2-oleoyl-sn-glycero-3-phosphocholine (POPC) using X-ray diffraction, Molecular Dynamics simulations, and electrochemical and leakage assays. The membrane surface attraction was varied by enriching the membrane with anionic phosphatidyl-L-serine residues. Membrane rigidity was at the same time controlled by inclusion of either POPS (1-palmitoyl-2-oleoyl-sn-glycero-3-phospho-L-serine), which preserves the membranes’ fluidity, or anionic lipids tails with saturated dimyristoyl tails (DMPS), which lead to denser acyl chain packing.

## Results

### X-ray diffraction

**Fig. 1:**
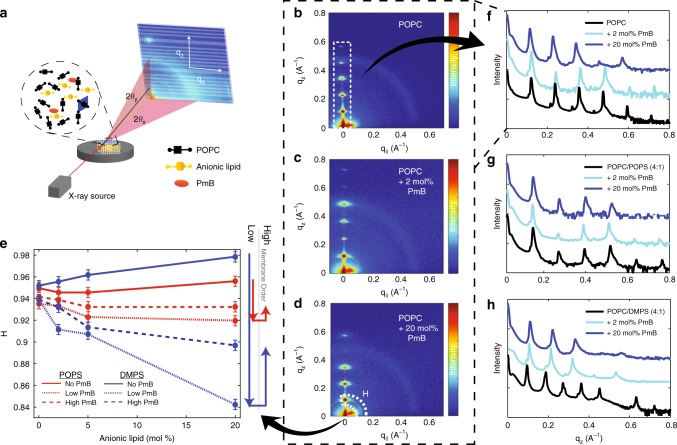
A schematic of the X-ray diffraction set-up, and a lipid unit cell (blue) as seen from the top, is shown in **a**. 2D intensity maps from X-ray diffraction of POPC (**b**), with 2 mol% PmB (**c**) or 20 mol% PmB (**d**). Herman’s orientation function as calculated from the radial integration of the first order [100]_*L*_ peaks are shown in **e**. The out-of-plane scattering by integrating along *q*_||_ = 0 Å^−1^ is shown in (**f**–**h**) for membranes of POPC (**f**), POPC/POPS (4:1) (**g**), and POPC/DMPS (4:1) (**h**), each with +2 mol% and +20 mol% PmB. (Error bars follow Poisson Statistics $\left( {\sqrt N } \right)$ but are smaller than point size.)

Highly-oriented lipid bilayers of POPC enriched with either POPS or DMPS at low-concentration and high-concentration regimes of PmB (2 and 20 mol%) were prepared, as described in the Methods section. The experimental set-up is shown in Fig. 1a. Each sample was equilibrated for 3 h before and maintained at 97% relative humidty (RH) during the measurement. Figure 1b–d shows two-dimensional diffraction patterns of these bilayers. A single series of Bragg peaks along the out-of-plane, *q*_*z*_, axis is indicative of highly-oriented multi lamellar membranes. The lamellar spacing *d*_*z*_ (membrane width + hydration water layer thickness) was determined from the peak positions and is plotted in Supplementary Figure 1. While *d*_*z*_ was found to increase with the addition of PmB in POPC/POPS membranes, the lamellar spacing decreases in POPC/DMPS membranes.

By radially integrating around the [100]_*L*_ Bragg peak, Herman’s orientation function, $H = \frac{3}{2} < {\mathrm{cos}}^2(\psi ) > - \frac{1}{2}$, was used to determine membrane orientation. Perfectly oriented membranes show *H* = 1 while randomly oriented membranes would result in *H* = 0.25. As shown in Fig. 1e, POPC bilayers show *H* ~ 0.95. While the addition of up to 20 mol% of POPS did not affect *H* within the experimental error, addition of 20 mol% DMPS increased *H* to 0.98. The addition of PmB caused a decrease in *H* in both POPS-enriched and DMPS-enriched membranes. The magnitude of these changes is greater in DMPS-enriched bilayers indicating strong membrane bending in the presence of PmB.

**Fig. 2:**
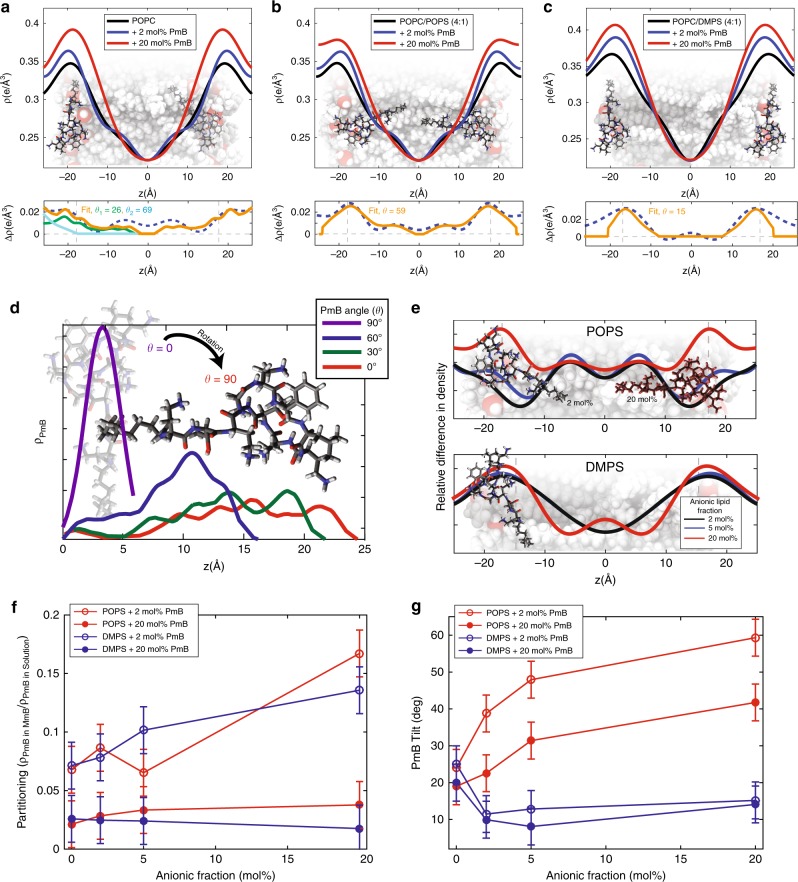
Electron density profiles, *ρ*(*z*), for membranes of POPC (**a**), POPC/POPS (4:1) (**b**), and POPC/DMPS (4:1) (**c**) with +2 and +20 mol% PmB. Difference profiles between +2 mol% PmB and pure bilayer are shown below. PO lipids are shown in white, DM lipids in grey in the background bilayers. In each subplot, orange corresponds to total fit, while blue and green correspond to multiple PmB population fits. **d** The theoretical electrical density of PmB. **e** The relative density of PmB along the bilayer normal in POPS-enriched and DMPS-enriched membranes at varying anionic lipid fractions. **f** Partitioning of PmB into the membrane unit cell versus in bulk solution, and **g** tilt of PmB with respect to the membrane axis. Fit parameters and tilts are given in Supplementary Table 1. (Error bars display the errors received from the fits)

Reflectivity curves were integrated from the two-dimensional data and are shown in Fig. 1f–h. Corresponding electron densities, *ρ*(*z*), were calculated from the integrated peak intensities, as shown in Fig. 2a–c. The position of the PmB molecules can be determined from these densities. Difference profiles were calculated as Δ*ρ*(*z*) = *ρ*(*z*)_Mem+PmB_ − *ρ*(*z*)_*Mem*_ and assigned to the density of PmB in the bilayer, shown in Fig. 2d.

By modeling the electron density of a PmB molecule in different orientations (in Fig. 2d), the exact location and orientation of the molecules in the bilayers can be determined by fitting the calculated density to the difference profiles, as shown in Fig. 2e. In POPC/POPS membranes, the PmB molecules were found to penetrate the bilayers while in POPC/DMPS bilayers PmB lies flat on the membranes. The relative electron density points to increased density in the bilayer center in the presence of DMPS at high PmB concentrations. We speculate that DMPS generally aligns its head groups with the phosphate plane of the POPC lipids. The association of PmB with the charged DMPS lipids through electrostatic interactions may push the shorter DMPS chains towards the bilayer core, leading to the increase in density in the bilayer center. We note that the increased electron density may, however, also be indicative of a small population of PmB molecules residing in the bilayer center.

Membrane partitioning was calculated by dividing the experimentally-determined number of membrane-embedded PmB molecules divided by molecules outside the bilayers (Fig. 2f). In POPC bilayers, we observe a 3-fold increase in PmB partitioning from 2 to 20 mol%. Increasing the POPS molar fraction at both low and high PmB concentrations shows increased partitioning. However, increasing the DMPS molar fraction at both low and high concentrations shows no difference in PmB insertion at 2, 5, and 20 mol% DMPS within the experimental errors.

The molecule tilt is shown in Fig. 2g. *θ* = 90° corresponds to PmB molecules perfectly parallel with the lipid normal, and *θ* = 0^°^ corresponds to a PmB perpendicular to the lipid normal. With increasing POPS concentration, PmB orients parallel to the membrane normal, while in DMPS-enriched bilayers, PmB was found to orient perpendicular to the membrane normal at 2 mol% PmB.

### Molecular Dynamics Simulations

All-atom membrane simulations were conducted using the SLipids force field in the GROMACS 5.1.4. Molecular Dynamics package. 128-lipid bilayers of POPC with either POPS or DMPS at different lipid ratios were prepared using MemGen^22^, as described in the Methods section. Simulations were equilibrated with an NVT/NPT ensemble and simulated for 250 ns. The final trajectories were extracted and 2 molecules of PmB were added to the water layer to mimic low-concentration experiments. The simulations were then re-requilibrated and run for at least another 100 ns. In total, this work includes ~7 μs of all-atom simulations with ~30,000 atoms per system. Details on simulated systems are given in Supplementary Table 2.

**Fig. 3:**
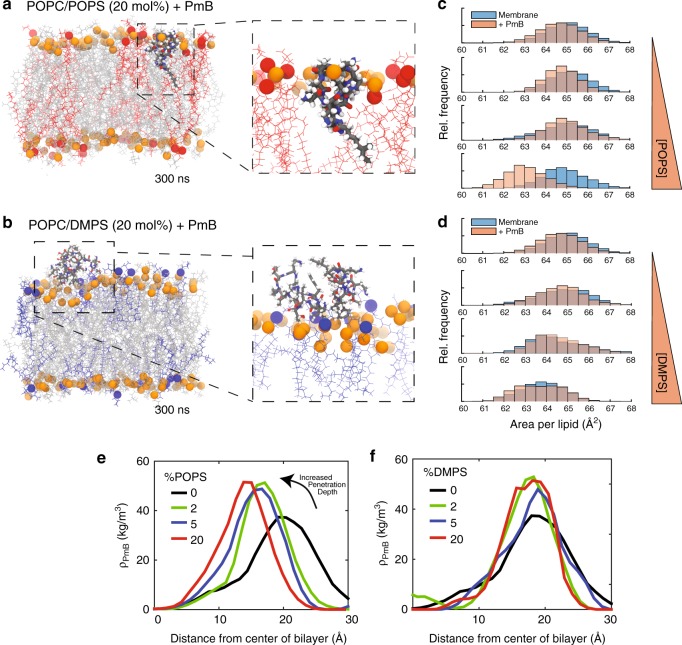
Snapshots from Molecular Dynamics Simulations of membranes with **a** 20 mol% POPS (red) or **b** 20 mol% DMPS with PmB initially external to the bilayer. Area-per-lipid distributions for membrane systems with PmB from final 50 ns of all-atom simulations described with increasing **c** POPS and **d** DMPS. Mass density of PmB from initially inserted PmB in varying membrane systems of increasing **e** POPS and **f** DMPS. Simulation components are given in Supplementary Table 2

Snapshots from Molecular Dynamics Simulations are shown in Fig. 3a, b. PmB is highlighted in blue, phosphate head groups are highlighted in orange, POPS and DMPS are highlighted in red and blue, respectively. While the PmB molecules were found to penetrate the bilayers in POPC/POPS membranes, PmB localizes within the lipid head groups, only, in POPC/DMPS membranes, in agreement with the experiments. The denser lipid packing can be observed from the calculated area per lipid molecule. In POPS-doped bilayers, PmB induced a reduction in the area-per-lipid while no difference was observed in DMPS-doped bilayers, as shown in Fig. 3c, d. At 20 mol% POPS, the reduction in area-per-lipid was found to be 2.6 Å^2^.

**Fig. 4:**
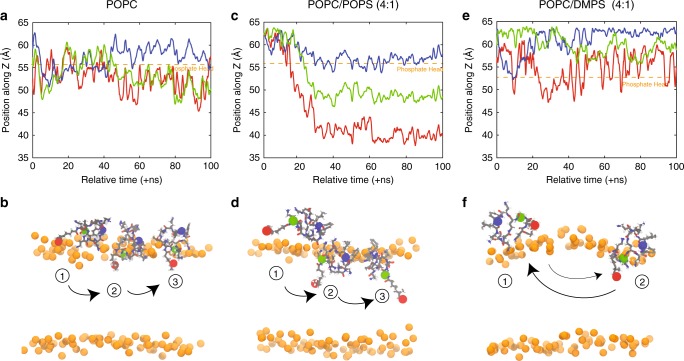
Insertion kinetics of individual molecules of PmB in **a**, **b** POPC, **c**, **d** POPC/POPS (4:1), and **e**–**f** POPC/DMPS (4:1). Line colors represent different atoms that correspond to the colored atoms in each PmB shown below. Insertion is shown over the 100 ns during which an event occurring

The position of PmB as a function of anionic lipid fraction is plotted in Fig. 3e, f. In agreement with the experiments, the PmB molecules penetrate the bilayers more deeply with increasing membrane charge in POPC/POPS membranes while the PmB molecules lay on the membrane surface in POPC/DMPS bilayers. The different insertion kinetics is shown in Fig. 4. While only the hydrophobic tail inserts in pure POPC membranes after 50 ns, full insertion is observed in POPC/POPS bilayers after about 30 ns of simulations. In POPC/DMPS membranes, the PmB molecules adhere to the membranes, but were not observed to penetrate fully into the bilayers.

PmB was also computationally titrated into the edge of the simulation box every 20 ns until a total PmB to lipid ratio of 20 mol%, corresponding to 10 PmB molecules was reached. PmB was found to form aggregates at high PmB concentrations on the bilayer. Single PmB molecules were found to penetrate the bilayer surface within the aggregate, as shown in Supplementary Figure 3.

### Electrochemical and leakage assays

To confirm the formation of defects in the membranes due to the addition of PmB, Cyclic Voltammetry (CV) measurements were done using a three-electrode electrochemical cell. The lipid membranes were applied to a working Au electrode. The microfabrication, lipid deposition, and electrochemical set up is detailed in the Methods Section. Briefly, POPC membranes with different molar fractions of either POPS and DMPS, doped with PmB, were deposited as a passivating layer on the working Au electrode following the same protocol from X-ray diffraction experiments, and placed in solution containing PmB as the analyte. The redox-active molecule ferrocyanide was used as a reporter and added to the buffer used during the electrochemical sensing experiments. The voltage on the working electrode was swept between 0 and 0.4 V, a range suitable for the reduction and oxidation of the reporter. When the membranes are intact, no current from the reduction or oxidation of the reporter is observed, as the lipid membrane prevents the transfer of electrons between solution and electrode surface. On the other hand, when the membrane is damaged, the aqueous buffer (and therefore the reporter) can access the surface of the electrode and current will appear in the form of cathodic or anodic peaks. The greater the amplitude of the redox peaks, the greater the membrane damage.

**Fig. 5:**
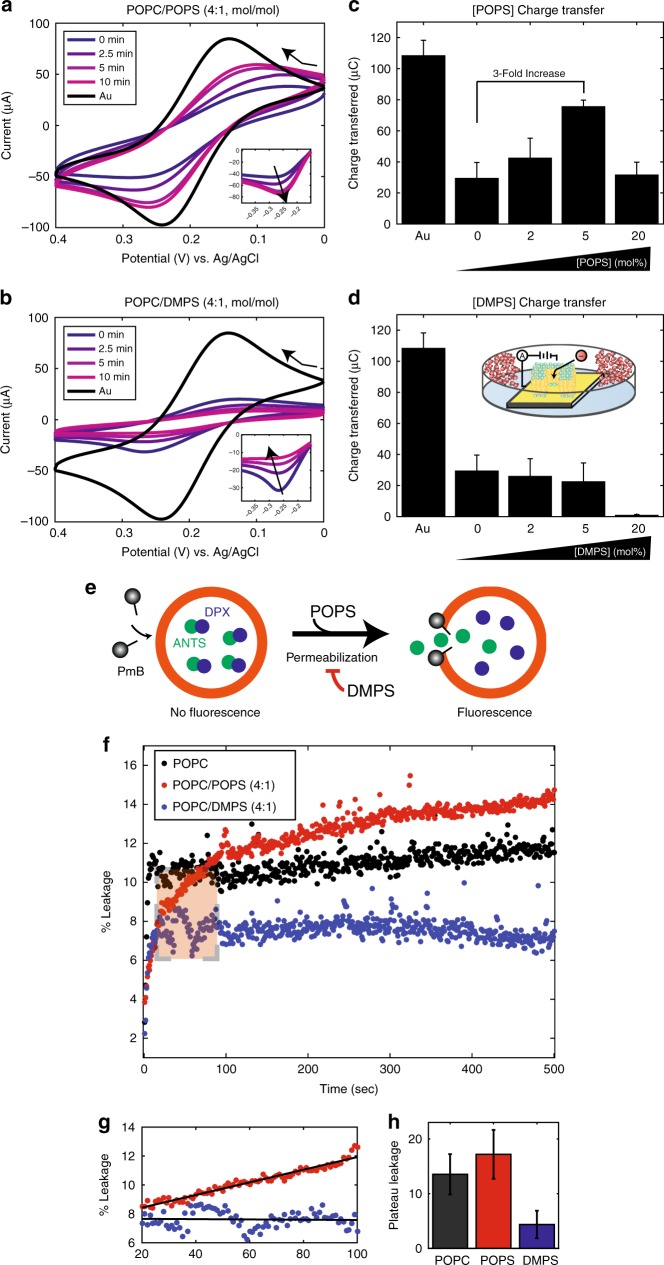
**a** CV curve of POPC/POPS over the course of 10 min until plateau is reached, and **b** charge transferred in μC as a function of POPS mol fraction. **c** CV curve for POPC/DMPS over ten minutes, and **d** charge transferred. Subplot shows schematic of three-electrode circuit, bent arrows indicate sweeping direction. **e** Schematic of leakage assays, and **f** fluorescence Leakage of POPC membranes with 20% POPS (red) and 20% DMPS (blue) over the first 500 s of an hour-long measurement. **g** Initial values and **h** equilibrium fluorescence values from highlighted box. Error bars represent standard deviation of values considered at equilibrium

PmB was added to the sensing solution, and the resulting voltammograms for POPC/DMPS (4:1) and POPC/POPS (4:1) are shown in Fig. 5a, b, respectively. Upon addition of PmB, voltammograms were recorded at 0, 2.5, 5, 10, and 20 min. In membranes with POPS, the amplitude of both peaks increases over time, suggesting membrane rupture occurs over time shown in the inset in Fig. 5a. In membranes with DMPS, the opposite effect is observed where the amplitude of the peaks is suppressed, suggesting membrane stabilization shown in the inset in 5b).

Due to the formation of membrane damage, i.e., pores, mass transport of the reporter to the electrode surface is diffusion dominant. Both convection and charge migration are minimized such that Randles-Sevcik equation may be used to accurately determine the charge transferred. To account for peak broadening, a non-faradaic offset was implemented to determine the peak current at all timepoints for the reduction peak, and from this, the charge transferred was calculated. Increasing the membrane surface charge with POPS-doped POPC membranes increased charge transfer by three-fold as seen in Fig. 5c. With increasing molar fraction of DMPS and constant PmB concentration, the current transfer decreases slightly with respect to a pure POPC membrane (Fig. 5d). We note that in both assays the highest (20 mol%) anionic fraction does not seem to follow the respective trend as lower membrane damage was observed at both 20 mol% POPS and DMPS. At 20 mol% of anionic lipids, it was found that POPS can adsorb ions from solution, which may be artificially affecting the charge transferred in our experiments at these high anionic fractions^23^.

PmB induced membrane damage was also checked using ANTS/DPX-loaded vesicles composed of POPC, POPC/POPS (4:1), and POPC/DMPS (4:1), as pictured in Fig. 5e. An optimal PmB concentration to use in solution, of 8.75 mg/mL, was determined by titration, as shown in Supplementary Figure 2. PmB was added to the solution, and leakage was observed for 1 h. As shown in Fig. 5f, PmB induces leakage from most to least in POPC/POPS>POPC>POPC/DMPS membranes. Figure 5g shows the initial 100 s in magnification and Fig. 5h plots the corresponding equilibrium fluorescence values. The presence of DMPS in the membrane reduced the leakage by three-fold and two-fold relative to POPS-doped, or pure POPC membranes, respectively, indicative of reduced membrane damage.

## Discussion

We find a two step model for lipopeptide insertion, where the lipopeptide first adheres to the charged residues in the lipid bilayer, and subsequently creates bending in the lipid bilayer. In the final stage, simulations show the lipopeptide to insert into the free space between lipids heads into the bilayer. Upon insertion, the curvature is relieved and the bilayer width accommodates the additional lipopeptide constituents, as shown at high PmB concentrations. The degree of membrane orientation is greatly increased in DMPS-enriched bilayers relative to POPS-enriched bilayers at low PmB concentrationsn. At high PmB concentrations, the presence of inserted PmB in the lamellar phase, rather than aggregated PmB, may lead to increased bilayer order. While in POPS-enriched bilayers, increased POPS lipid fractions reduce the tilt of the PmB within the bilayer and leads to increased penetration depth.

Di-aminobutyric acid residues on PmB have been reported to bind on Lipid A^15^. Here, we find that PmB is able to spontaneously insert into lipid bilayers without Lipid A in the system. These results are consistent with the notion that PmB insertion is electrostatically driven^3^. While Lipid A may accelerate the coupling of PmB-membrane complexes, the presence of specifically lipid A may not be a neccessary condition for PmB-induced membrane destabilization or cation displacement^24^.

From experiments and simulations, increasing the anionic fraction in POPC membranes by including POPS lipids led to an increase in membrane partitioning of PmB. The hydrophobic tail of the PmB molecule partitions deeper and aligns in the membrane the higher the membrane charge. When adding saturated chains (DMPS) to the POPC membrane, lipid packing increases (the area-per-lipid decreases) and PmB penetration drastically decreases. PmB preferably lies flat on the bilayers and does not penetrate. Partitioning of PmB led to membrane damage in POPC/POPS membranes, while the addition of DMPS as the anionic lipid led to a reduction in damage.

**Fig. 6:**
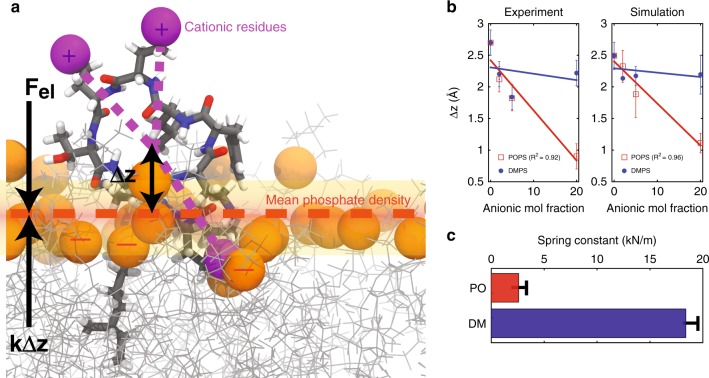
**a** Schematic for a model of PmB-induced membrane damage, where primary cationic residues are highlighted in purple. The phosphate density is shown in orange. The distance between the triangulated cationic residues and the phosphate density is defined Δ*z*. **b** Δ*z* calculated independently from experiments and simulations. The resulting membrane resistance to PmB-insertion is given by spring constant *k*, as calculated from experimental positions of PmB (shown in (**c**))

The two dominant mechanisms involved are the membrane attraction of PmB, which increases with membrane charge, and the insertion of the PmB molecules, which is impeded when the lipid packing is increased. One may assume a simple model, where PmB attraction is driven by the electrostatic interaction between the positive residues in PmB and the negative charges in the membranes. We can also assume that insertion of the lipophilic PmB residue into the membrane core is impeded by a force proportional to the insertion, *k*Δ*z*. *k* is a phenomenoligical spring constant that quantifies the stiffness of the membranes against insertion of PmB. The model is pictured in Fig. 6a.

The equilibrium between these two forces should determine the location of PmB in the different bilayers: *EQ*_PmB_ = *k*Δ*z*. The electric field of a charged plane can be written as $E = \frac{\sigma }{{2\varepsilon _0}}$ (with *ε*_0_ = 8.85 ×10^−12^ F/m and *Q*_PmB_ = # of charges of one PmB molecule). The membrane surface density depends on the anionic lipid fraction as *σ* = *Q*_mem_/*A*_mem_, where *Q*_mem_ = *n*_lipids_ × mol%, where *n*_lipids_ is the number of lipids. The membrane area for POPC/POPS membranes is then *A*_mem_ = *n*_lipids_*a*_*P*_. We did not observe a change in the area-per-chain as a function of POPS concentration within the resolution of our experiments and simulations. For POPC/DMPS bilayers, the area can be written as *A*_mem_ = *n*_lipids_ (100 − mol%) *a*_*P*_ + *n*_lipids_ mol% *a*_*D*_ ≈ *n*_lipids_ *a*_*P*_ with *a*_*P*_ = 64 Å^2^ the lipid area of POPC and *a*_*D*_ = 58 Å^2^ the area for DMPS^25,26^.

The insertion depth of PmB, Δ*z*, can then be written as

1 $${\mathrm{\Delta }}z = \frac{{Q_{PmB}\sigma }}{{2\varepsilon _0k}} = \frac{{Q_{PmB}}}{{2\varepsilon _0ka_P}} \times {\mathrm{anionic}}\,{\mathrm{mol}}\,{\mathrm{fraction}}.$$

In this model, the insertion depth of the PmB molecules is a linear function of the anionic fraction. The corresponding data from experiments and simulations are plotted in Fig. 6b and indeed show a linear behavior. The phenomenological spring constant *k* can be determined from the slope and is plotted in Fig. 6c. We determine a resistance of 2500 N/m for POPC/POPS membranes and a higher resistance of 18,000 N/m for POPC/DMPS. The spring constant for graphite for comparison is calculated to 27,000 N/m for the in-plane interaction, and 3.5 N/m for the much weaker out-of-plane interactions between graphite layers^27^. The force constants that we measure for the PmB insertion are, thus, about one order of magnitude weaker than a covalent bond, however, about 1000 times stronger than a hydrogen bond. We note that the approximations made in Eq. (1) neglect the fact that *k* is not constant but likely increasing with increasing anionic mol fraction: charge effects dominate at small anionic lipid fractions, while the ordering of saturated lipids dominate at large lipid fractions. However, the straight line approximation provides a satisfactory fit within the statistics of our data.

We note that the use of PE/PG lipids would be more representative of bacterial membranes. There were two factors that rationalized PC/PS lipids over PE/PG. First, at a neutral pH, PS lipids have a charge of −1 while PG is in a biased equilibrium between anionic and neutral form such that its charge is reduced^28^. In addition, in response to cell-penetrating peptides, researchers were unable to determine why POPG had a 3-fold increase in surface pressure in response to these peptides compared to both POPC and POPS in Langmuir trough experiments^29^. We therefore argue that PC/PS head groups are better suited to study the hydrophobic and electrostatic interactions in PmB action.

The results present evidence that acyl chain ordering has an effect on PmB-associated membrane damage. It was shown recently that cholesterol is able to also greatly reduce PmB-associated membrane damage^21^, which most likely points at a general trend that lipid packing is inversely correlated with PmB-associated damage. However, further work including also bacterial sterols is required to unambiguously clarify this relationship.

With the advent of colistin-resistance, first observed in 2016^7^, bacteria have been reported to be capable in changing membrane structure to resist antibiotics. In *mcr*-1 resistant bacterial strains, an additional phosphatidylethanolamine is attached to lipid A in the outer-bacterial membrane affecting membrane packing and surface charge. For instance, the resistance has been speculated to be due to an increased acyl packing, which would increase bilayer rigidity^13^.

As PmB is a cationic lipopeptide, the increase in lipid surface charge increases the coulombic attraction between peptide and membrane surface. The addition of a phosphatidylethanolamine to lipid A in the resistant bacterial strain reduces the net charge of a single lipid A from −1.5 to −1.0 e^−^ per lipid A^30,31^. Since bacterial species have a wide range in the fraction of lipid A fraction in the outer-bacterial membrane, a proper estimate for the anionic charge difference is difficult to obtain, but species such as *E. coli* have a lipid A fraction of 70% in the outer membrane, thus an *mcr*-1 phenotype would reduce the charge attraction by 23%^15,32^.

Our results and phenomenological model suggest that the *mcr*-1 resistant membranes can reduce PmB insertion into the bilayers by reducing surface charge density and increasing acyl packing. Thus, for *mcr*-1 expressing *E. coli*, assuming complete Lipid A modification, one can expect that the penetration depth would be reduced by 1.52 Å from electrostatic attractions and up to 11-fold greater membrane resistance. This may explain the four to 16-fold increased resistance in *mcr*-1 expressing *E. coli*^33^.

**Fig. 7:**
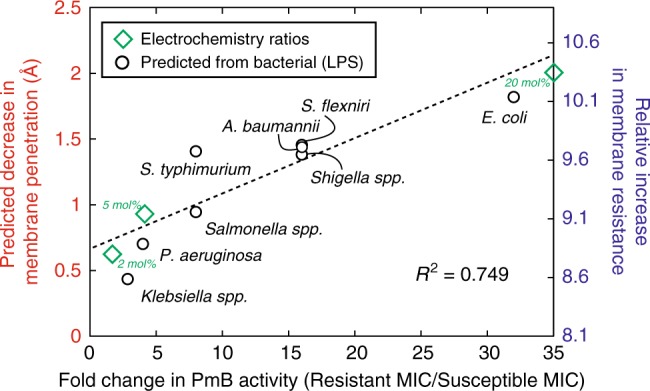
(Black) Fold increase in resistance for reported polymyxin-resistant Gram-negative species with assumed change in polymyxin penetration and membrane resistance from reported lipid A content. (Green) Fold-change resistance from POPS/DMPS values for membrane damage from electrochemistry compared to determined changes in penetation depth. This model provides a reasonable description of the data, as indicated by the linear fit

By examinging the lipid A (or LPS) fraction in gram-negative bacterial species, with observed polymyxin resistance, we estimated the fold-change in resistance in Fig.7. We first extracted MIC values from literature, as described in Supplementary Table 3, and then either determined, or predicted, the fold-change in resistance with the reference CLSI clinical resistance breakpoint of 4 μg/mL and the MIC of non-resistant strains. By assuming that there is a $\frac{1}{3}$ reduction in membrane charge, associated with the addition of phosphoethanolamine, we correlated the predicted fold-change in resistance with the change in membrane charge, and thus the membrane rigidity, from the initial concentration of lipid A in the bacterial membrane. The used MICs are given in the Supplementary Information for PmB with the following bacterial strains: *A. baumani, P. aeruginosa, E. coli, Klebsiella spp., Salmonella spp., Shigella spp., S. typhimurium, S. flexneri*. Both mcr-1 and prm activation can remove the presence of a free phosphate on the lipid A core, and are considered. To complement this, the fold change in electrochemistry was determined by *Fold Increase*(*x*) = *CT*_*POPC*/*xPOPS*_/*CT*_*POPC*/*xDMPS*_), where *CT* is charge transferred, and *x* is the anionic molar fraction in complement to diffraction results.

As shown in Fig. 7, bacterial species with a greater lipid A content are expected to have a greater fold-change in resistance to PmB. As indicated by th linear fit, this model provides a reasonable description of the data. We note that the model membranes used in this work do not represent many common bacterial features, such as membrane assymetry or diverse compositions; however, our simple phenomenological model may be used to explain which species will show a greater fold-increase in resistance based on membrane charge and corresponding rigidity.

In summary, by combining X-ray scattering, Molecular Dynamics simulations, computational titration, electrochemistry, and fluorescence leakage, we investigated the mechanisms of PmB-induced membrane damage in vitro. PmB first adsorbs to the bilayer surface, which induces membrane disorder as shown in diffraction, prior to inserting, and then orders in concordance with lipid packing subsequent to insertion. We varied lipid packing and increased membrane tail packing to PmB-induced damage to understand the phenotypic attributes of *mcr*-1 resistance bacteria. Our results show that increased surface charge increases the penetration depth and reduces PmB tilt within the bilayer, promoting membrane damage; however, an increase in tail packing can inhibit membrane insertion.

From a simple phenomenological model, we suggest that electrostatic interactions between PmB and the membrane surface promotes PmB insertion, while membrane packing and rigidity gives rise to membrane resistance. Altogether, our results suggest that the *mcr*-1 resistant membrane have reduced surface charge and increased acyl packing, which may be preventing both PmB adsorption to the bacterial surface and insertion into the bilayer, respectively.

## Methods

### X-ray scattering experiments

Highly-oriented multi lamellar membranes were prepared on a single-side polished silicon wafer^34^. 1-palmitoyl-2-oleoyl-sn-glycero-3-phosphocholine (POPC, Avanti), 1-palmitoyl-2-oleoyl-sn-glycero-3-phospho-L-serine (POPS, Avanti), and 1,2-dimyristoyl-sn-glycero-3-phospho-L-serine (DMPS, Sigma) were mixed with PmB (Sigma) at 2 and 20 mol% concentrations in 2,2,2-trifluoroethanol:Chloroform (1:1, vol/vol) mixture at a soluton concentration of 18 mg/mL. The wafers were sonicated in 1,2-dichloromethane for 30 min, and then rinsed with alternating methanol and 18.2 MΩ ⋅ cm water. The wafers were dried, and 75 μL of solution was deposited. After drying, the samples were placed in a vacuum for 24 h at 310 K to allow for trace solvent evaporation and annealing. Samples were then hydrated in a closed chamber at 97% RH with a separate K_2_SO_4_ saturated solution for 48 h prior to scanning.

X-ray diffraction data was obtained using the Biological Large Angle Diffraction Experiment (BLADE) at McMaster University. BLADE uses a 9 kW (45 kV, 200 mA) CuKα rotating anode at a wavelength of 1.5418 Å using a Rigaku HyPix-3000 2D semiconductor detector with an area of 3000 mm^2^ and 100 μm pixel size^35^. All samples were prepared and measured in replicates to check for consistency. As is common practice in diffraction studies, data shown are the results of single measurements. The good statistics in such measurements is the result of the large spatial and temporal averages, which include a large number of molecules and ensembles.

### Out-of-plane structure and electron densities

The relative electron density, *ρ*(*z*), can be approximated by a one-dimensional Fourier analysis,

2 $$\rho (z) = \frac{2}{{d_z}}\mathop {\sum}\limits_{n = 1}^N \sqrt {I_nq_n} \nu _n\,{\mathrm{cos}}\,\left( {\frac{{2\pi nz}}{{d_z}}} \right).$$

*N* is the highest order of the Bragg peaks observed in the experiment. The integrated peak intensities, *I*_*n*_, are multiplied by *q*_*n*_ to receive the form factors, *F*(*q*_*n*_)^36,37^. The bilayer form factor *F*(*q*_*z*_), which is in general a complex quantity, is real-valued in the case of centro-symmetry. The phase problem of crystallography, therefore, simplifies to the sign problem *F*(*q*_*z*_) = ±|*F*(*q*_*z*_)| and the phases, *v*_*n*_, can only take the values ±1. The phases *v*_*n*_ are needed to reconstruct the electron density profile from the scattering data following Eq. (2), and are well-defined by previous literature. The out-of-plane diffraction data is available in Supplementary Figure 4. When the membrane form factor, F(*q*_*z*_) is measured at several *q*_*z*_ values in a continuous fashion, *T*(*q*_*z*_), which is proportional to *F*(*q*_*z*_), can be fit to the data:

3 $$T(q_z) = \Sigma _n\sqrt {I_nq_n} \mathrm{sin}c\left( {\frac{1}{2}d_zq_z - \pi n} \right).$$

In order to determine the phases quantitatively, the form factor has to be measured at different *q*_*z*_ values using the so-called swelling technique or by measuring the bilayer at different contrast conditions when using neutron diffraction. In this work, the phases, *v*_*n*_, were assessed by fitting experimental peak intensities and comparing them to the analytical expression for *T*(*q*_*z*_) in the above equation. An array of phases [−1 −1 1 −1 −1] was used for all samples.

*ρ*(*z*) is initially calculated on an arbitrary scale, they are then scaled based on the protocol established in our previous work^38^. The curves are scaled until the total number of electrons within the lipid unit cell across a membrane leaflet, $e^ - = A_L{\int}_0^{d_z/2} \rho (z)d_z$ agrees with the total number of electrons expected based on the sample composition. The area per lipid (*A*_*L*_) was set to 64.3 Å^2^ for a hydrated POPC bilayer^39^.

*ρ*(*z*) was scaled by the number of electrons per unit cell, including the electron contributions from the membrane lipids, PmB, and water, while the bilayer core, *z* = 0 Å was fixed at 0.22 *e*^−^/Å^3^ to represent terminal methyl groups of the lipid acyl chain.

### Cyclic voltammetry

Cyclic voltammetry (CV) was performed using a 600 E Electrochemical Workstation (CH Instruments, Austin, TX). A three-electrode configuration was used to sense for K_4_[Fe(CN)_6_] in the working solution. The reference electrode used was a standard Ag/AgCl electrode (CH Instruments), Pt auxiliary electrode (CH Instruments), and a flat Au working electrode. The CV sensing parameters were −0.4–0 V sensing sweep range, +0.1 V/s scan rate, and duration of 10 segments. Briefly, a 20 mL sensing solution was pipetted into a glass dish where the Ag/AgCl reference electrode and the Pt auxiliary electrode were immersed and connected to the potentiostat. The Au working electrode was rinsed with 18.2 MΩ cm water before immersion into the sensing solution and also connected to the potentiostat. The CV scans were taken at a temperature of 25 °C, every 1 min interval until the reduction peak current plateaued for each measurement. Scans were taken to observe the behaviour of this peak current, and it was observed that the peak current generally plateaued after 10 min. Once the electrodes established a stable plateau, they were rinsed with IPA, EtOH, and 18.2 MΩ cm water respectively to remove any residual lipids and PmB. They were then placed back into the sensing solution and CV scanned for 10 segments to ensure recoverability of the Au working electrode.

The working solution was prepared by adding ~0.2 g of K_4_[Fe(CN)_6_] salt (Sigma-Aldrich) to 100 mL of 1 × PBS buffer (BioShop Canada, Burlington, Ontario, Canada) solution. To this, 50 mg of Polymyxin-B (Sigma-Aldrich) was added to make a final sensing solution of 5 mM K_4_[Fe(CN)_6_] 3H_2_O and 0.5 mg/mL PmB in 1 × PBS buffer.

### Fluorescence leakage assay

Liposome preparation: Lipids used were purchased from Avanti, and used without further purification. Lipid films were prepared by evaporating the chloroform dissolved lipids under an argon gas stream. They were dried further in a vacuum chamber overnight. After drying, the films were rehydrated in PBS and then vortexed. The liposome solution was then freeze-thawed 10 times using a liquid nitrogen and a water bath 10X to make the liposomes unilamellar. Lastly, the solution was extruded using a 100 nm diameter pore membrane using the Avanti Mini-Extruder 11X to obtain a more monodisperse liposome preparation. The liposome solution was then transferred to a vial, topped off with argon gas, wrapped in parafilm, and stored at 4 °C.

Membrane permeabilization assay: Membrane permeabilization was measured through a dye-release assay. Liposomes were prepared as described above with ANTS (8-Aminonaphthalene-1,3,6-Trisulfonic Acid, Disodium Salt) and DPX (p-Xylene-Bis-Pyridinium Bromide) (Molecular Probes) added to the PBS. Following extrusion, liposomes were passed through a 10 cm Sepharose CL-B2 column to remove non-encapsulated dye. Data was measured using a PTI Quantamaster fluorometer (Photon Technologies International) at an excitation wavelength of 355 nm (6 nm bandwidth) and an emission wavelength of 520 nm (12 nm bandwidth). The reaction was stirred at 100 rpm and conducted at 23 °C. Measurements were taken for up to 1 h. Background measurement of the liposome solution was conducted prior to each reaction. After reaction equilibration, Triton X 100 was added to allow for total dye release. Data was normalized using the following equation:

4 $${\mathrm{ANTS}}\,{\mathrm{Release}}\,{\kern 1pt} (\% ) = \frac{{F - F_{BG}}}{{F_{FR} - F_{BG}}} \times 100,$$

where *F* is fluorescence, and *BG* and *FR* refer to background and full release respectively.

### Molecular dynamics simulations

All simulations were run in-house on a GPU accelerated workstation, using the GROMACS 5.1.2 Molecular Dynamics package using the SLipids force field and the SPC water model. Fourteen systems of membranes were generated with MemGen according to Table S2. The lipid topologies for POPC, POPS, and DMPS were taken from Jämbeck et al.^40–42^. The PmB topology was generated using the Amber 14 program Antechamber, with partial charges and force field parameters based in the General Amber Force Field (GAFF). The SPC water model was used for solvation. The topology file is available from the author (MCR) upon request. All simulations used a 2 fs time step, a periodic boundary condition applied to all directions, the particle-mesh Ewald method to solve for long-range electrostatics, a short-range van der Waals cutoff of 1.2 nm, and the LINCS algorithm to determine bond constraints. Temperature coupling was controlled using a Nose-Hoover thermostat at 28 °C (*τ* = 0.5 ps) and pressure was kept at 1.0 bar using Parrinello-Rahman semi-isotropic weak coupling (*τ* = 1 ps). All systems were charge neutralized by the addition of sodium counterions.

Fourteen systems were prepared to model the two possible states of PmB in the membrane, at either low PmB concentrations (*n*_PmB_ = 2) or high PmB concentrations (*n*_PmB_ = 10). In either model, the membrane was depleted of water and a Monte-Carlo algorithm was used to insert PmB into available areas external to the bilayers, after which the system was resolvated to 25 waters-per-lipid. The systems were then charge neutralized based on the number of anionic lipids and PmB. The system was then simulated at 303 K and a pressure of 1 bar as according to Table S2. The last 20 ns of simulation were used for analyses to ensure that systems had fully equilibrated before quantitative data collection was performed unless otherwise specified.

For experimentally-determined simulations, where the PmB was inserted into the lipid bilayer, one lipid was removed from a generated 128-lipid patch from each leaflet. Then, 2 PmB molecules were inserted into these free volumes, and the initial tilt of the artificially inserted PmB was set to that as determined by experiment. The simulations were then run for 100 ns, unrestrained, with the same parameters provided above. The simulations resulted in a total of 7 μs of all-atom simulated time in replicate. Simulations were conducted where PmB was started in the middle of the water layer in duplicate, however, the time required for reliable insertion was inaccessible by the simulations in all conditions. This is a common situation in all-atom Molecular Dynamics simulations. We, therefore, conducted simulations with PmB inserted into the bilayer based on the experimentally-determined positions to sample PmB in its most probable states. Table S2 provides details on each simulated system in the Supplementary Information.

### Code availability

Computer code generated during the current study is available from the corresponding author on reasonable request.

### Reporting summary

Further information on experimental design is available in the Nature Research Reporting Summary linked to this article.

## Supplementary information

Supplementary Information

Description of Additional Supplementary Files

Supplementary Data 1

Supplementary Data 2

Supplementary Data 3

Reporting Summary

## Data Availability

The datasets generated and analysed during the current study are included in this published article and its Supplementary Information file or provided for download as Supplementary Data Files.

## References

[1] Laxminarayan R (2013). Antibiotic resistancethe need for global solutions. Lancet Infect. Dis..

[2] Baker S, Thomson N, Weill FX, Holt KE (2018). Genomic insights into the emergence and spread of antimicrobial-resistant bacterial pathogens. Science.

[3] Velkov T, Thompson PE, Nation RL, Li J (2009). Structure- activity relationships of polymyxin an-tibiotics. J. Med. Chem..

[4] Brogden KA (2005). Antimicrobial peptides: pore formers or metabolic inhibitors in bacteria?. Nat. Rev. Micro-Biol..

[5] Brochado, A. R. et al. Species-specific activity of antibacterial drug combinations. *Nature* **559**, 259–263 (2018). 10.1038/s41586-018-0278-9 PMC6219701 29973719

[6] Sprenger M, Fukuda K (2016). New mechanisms, new worries. Science.

[7] Liu YiY (2016). Emergence of plasmid-mediated colistin resistance mechanism mcr-1 in animals and human beings in china: a microbiological and molecular biological study. Lancet Infect. Dis..

[8] Wang R (2018). The global distribu-tion and spread of the mobilized colistin resistance gene mcr-1. Nat. Commun..

[9] Liu, Y-Y. et al. Structural modification of lipopolysaccharide con-ferred by mcr-1 in gram-negative eskape pathogens. *Antimicrob. Agents Chemother*. **61**, e00580-17 (2017). 10.1128/AAC.00580-17 PMC5444183 28373195

[10] Khakbaz P, Klauda JB (2015). Probing the im-portance of lipid diversity in cell membranes via molecular simulation. Chem. Phys. Lipids.

[11] McPhee JB, Lewenza S (2016). From pigs to patients: transmissible, single gene-mediated resistance to colistin. J. Med. Microbiol. Diagn..

[12] Guo L (1998). Lipid a acylation and bacterial resistance against verte-brate antimicrobial peptides. Cell.

[13] Rice A, Wereszczynski J (2018). Atomistic insights into the unique roles of lipopolysaccharide modifications in strengthening bacterial outer membrane defenses. BioPhys. J.

[14] Evans ME, Feola DJ, Rapp RP (1999). Polymyxin b sulfate and colistin: old antibiotics for emerging multiresistant gram-negative bacteria. Ann. Pharmacother..

[15] Berglund NA (2015). Interaction of the antimicrobial peptide polymyxin b1 with both membranes of *E. coli*: a molecular dynamics study. PLoS Comput. Biol..

[16] Kanazawa K (2009). Contribution of each amino acid residue in polymyxin B3 to antimicrobial and lipopolysaccharide binding activity. Chem. Pharm. Bull..

[17] Morrison DC, Jacobs DM (1976). Binding of polymyxin B to the lipid a portion of bacterial lipopolysac-charides. Immunochemistry.

[18] Rosenthal KS, Storm DR (1977). Disruption of the escherichia coli outer membrane permeability barrier by immobilized polymyxin. J. Antibiot..

[19] Clausell A (2007). Gram-negative outer and inner membrane models: insertion of cyclic cationic lipopeptides. J. Phys. Chem. B.

[20] Hale JDF, Hancock REW (2007). Alternative mechanisms of action of cationic antimicrobial peptides on bacteria. Expert Rev. Anti-Infect. Ther..

[21] Khondker A (2017). Membrane cholesterol reduces polymyxin B nephrotoxicity in renal membrane analogs. Biophys. J.

[22] Knight CJ, Hub JS (2015). Memgen: a general web server for the setup of lipid membrane simulation systems. Bioinformatics.

[23] Jurkiewicz P, Cwiklik L, Vojtfskova A, Jungwirth P, Hof M (2012). Structure, dynamics, and hydration of popc/pops bilayers suspended in NaCl, KCl, and CsCl solutions. Biochim. Biophys. Acta (BBA)-Biomembr..

[24] Velkov T, Roberts KD, Nation RL, Thompson PE, Li J (2013). Pharmacology of polymyxins: new insights into an oldclass of antibiotics. Future MicroBiol..

[25] Petrache HI (2004). Structure and fluctuations of charged phos-phatidylserine bilayers in the absence of salt. Biophys. J.

[26] Jo S, Lim JB, Klauda JB, Im Wp (2009). Charmm-gui membrane builder for mixed bilayers and its application to yeast membranes. Biophys. J.

[27] Rheinstadter MC, Schmalzl K, Wood K, Strauch D (2009). Protein-protein interaction in purple membrane. Phys. Rev. Lett..

[28] Marsh D (1990). CRC Handbook of Lipid Bilayers.

[29] Alhakamy NA, Elandaloussi I, Ghazvini S, Berkland CJ, Dhar P (2015). Effect of lipid headgroup charge and pH on the stability and mem-brane insertion potential of calcium condensed gene com-plexes. Langmuir.

[30] Nikaido H (2003). Molecular basis of bacterial outer mem brane permeability revisited. Microbiol. Mol. Biol. Rev..

[31] Olaitan AO, Morand S, Rolain JM (2014). Mechanisms of polymyxin resistance: acquired and intrinsic resistance in bacteria. Front. Microbiol..

[32] Luckey, M. Membrane structural biology: with biochemical and biophysical foundations (Cambridge University Press, Cambridge, 2014).

[33] Sieron, A. Characterization and high-throughput screening of the polymyxin resistance enzyme MCR-1, Ph.D. thesis, https://macsphere.mcmaster.ca/handle/11375/22269 (McMaster University, 2017).

[34] Pabst G, Kucerka N, Nieh MP, Rheinstadter MC, Katsaras J (2010). Applications of neutron and X-ray scattering to the study of biologically relevant model membranes. Chem. Phys. Lipids.

[35] Khondker A, Malenfant DJ, Dhaliwal AK, Rheinstadter MC (2018). Carbapenems and lipid bilayers: localization, partitioning, and energetics. ACS Infect. Dis..

[36] Nagle JF, Wiener MC (1989). Relations for lipid bilayers. Connection of electron density profiles to other structural quantities. Biophys. J.

[37] Barrett MA (2012). Interaction of aspirin (acetylsalicylic acid) with lipid membranes. PLoS ONE.

[38] Alsop, R. J., Khondker, A., Hub, J. S. & M. C. Rheinstadter. The lipid bilayer provides a site for cortisone crystallization at high cortisone concentrations. Sci. Rep. **6**, 22425 (2016). 10.1038/srep22425 PMC4776104 26936102

[39] Klauda JB, Kucerka N, Brooks BR, Pastor RW, Nagle JF (2006). Simulation-based methods for interpreting X-ray data from lipid bilayers. Biophys. J..

[40] Jaambeck JPM, Lyubartsev AP (2012). Derivation and systematic validation of a refined all-atom force field for phosphatidylcholine lipids. J. Phys. Chem. B.

[41] Jaambeck JPM, Lyubartsev AP (2012). An extension and further validation of an all atomistic force field for biological membranes. J. Chem. Theory Comput..

[42] Jambeck JPM, Lyubartsev AP (2012). Another piece of the membrane puzzle: extending slipids further. J. Chem. Theory Comput..

