# Supplementary material for: Membrane charge and lipid packing determine polymyxin-induced membrane damage
Source: Commun Biol. 2019 Feb 18;2:67. doi: 10.1038/s42003-019-0297-6 (PMC6379423; doi:10.1038/s42003-019-0297-6)
Supplement: Supplementary file 1 — Supplementary Information [file 42003_2019_297_MOESM1_ESM.pdf]

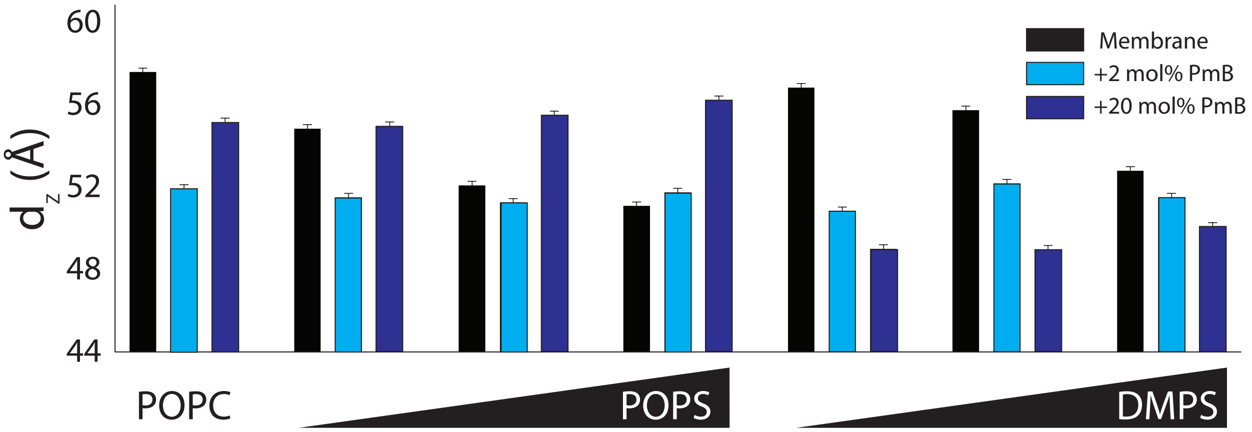

FIG. Supplementary Figure 1. Lamellar spacing, as calculated from $d_z = \frac{2\pi}{q_z}$, from out-of-plane scattering from x-ray diffraction.

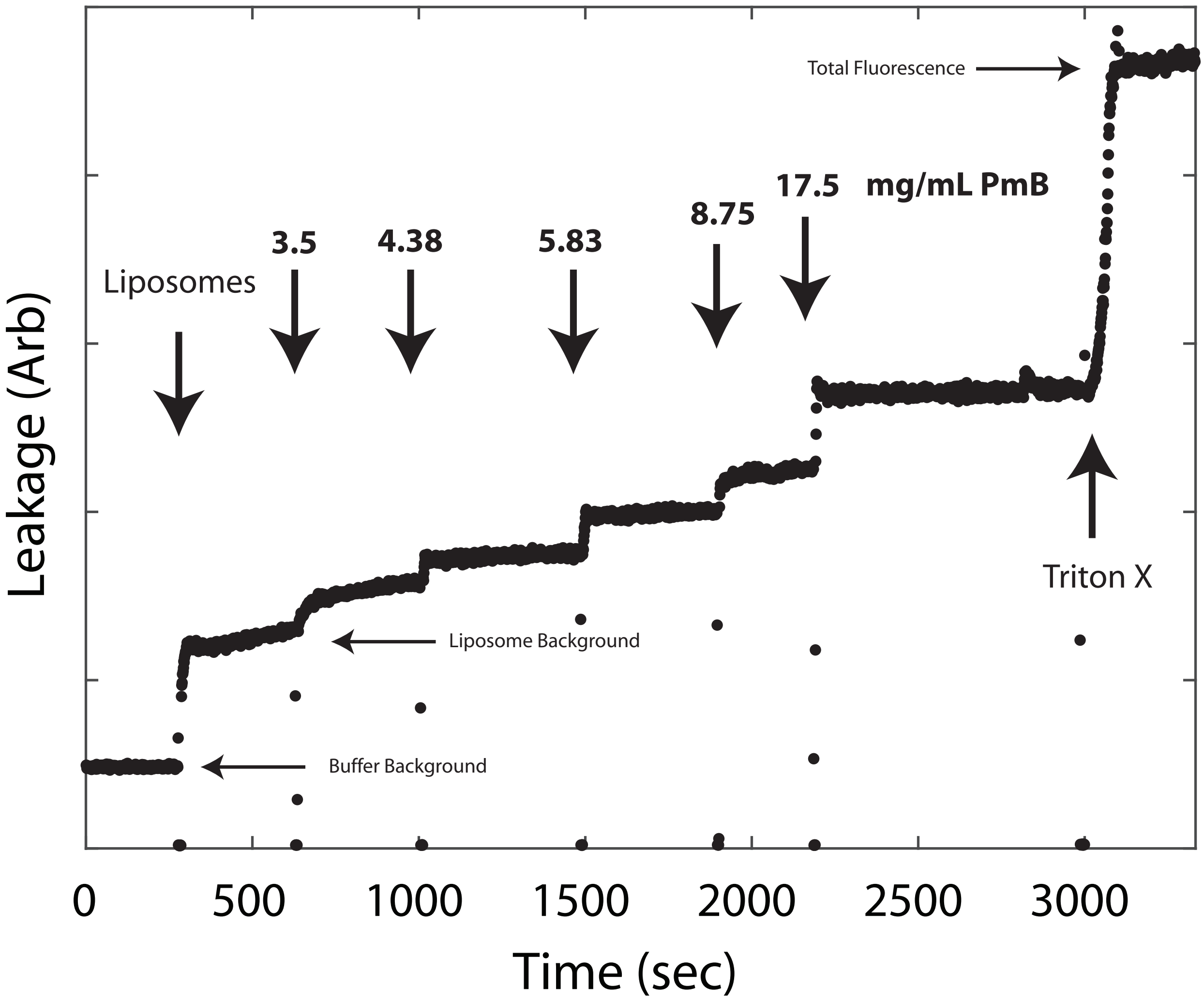

FIG. Supplementary Figure 2. Titration of PmB in liposome solutions to determine optimal concentration for use in fluorescence leakage experiments.

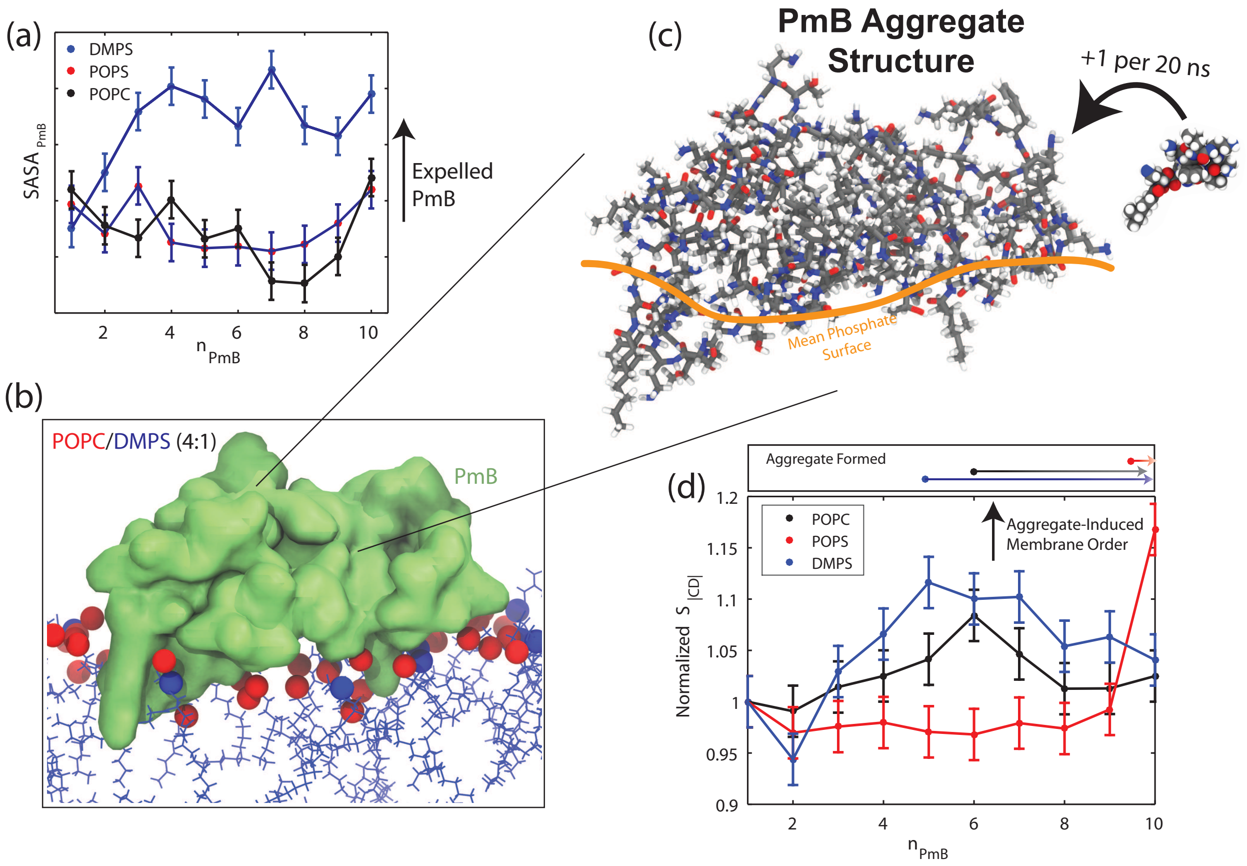

FIG. Supplementary Figure 3. (a) Solvent-accessible surface area for PmB in each titration. (b) Titration Simulation snapshot from POPC/DMPS (4:1) with 10 PmB shown in green, POPC lipids are not shown for clarity. (c) Aggregate structure of PmB. (d) Normalized deuterium order-parameter, and suggested aggregation timepoints from S_{CD}.

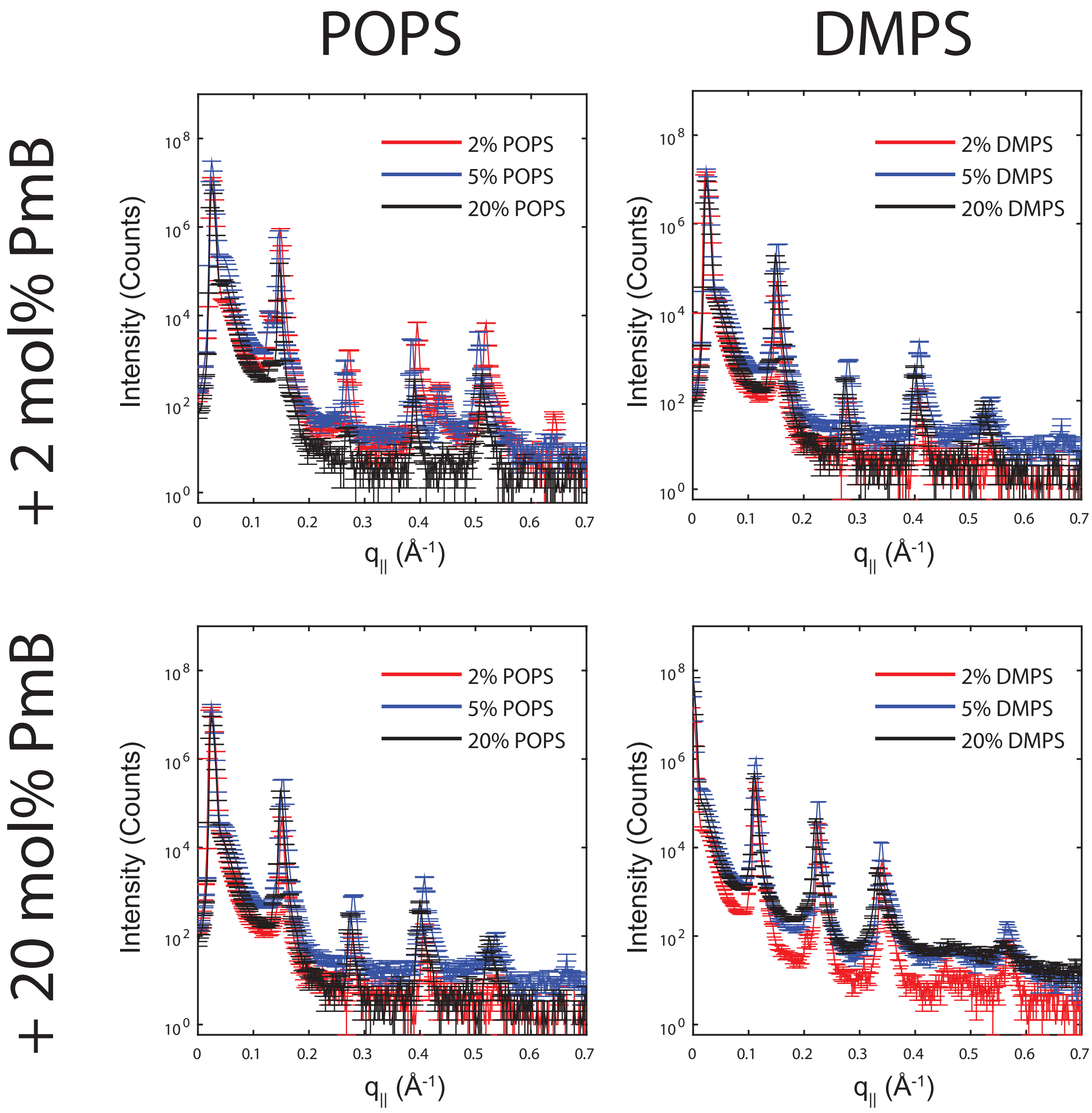

FIG. Supplementary Figure 4. X-ray diffraction data for each sample studied in this work along the out-of-plane axis. The two-dimensions diffraction data is available from the authors on request (AK, MCR).

PmB Regime	[DMPS] (mol%)	Head	Tail	d_{PmB} along Z
Low	2	24.41	4.81	19.60
	5	24.34	4.84	19.50
	20	24.8	4.882	19.92
High	2	25.01	5.31	19.70
	5	25.64	5.84	19.80
	20	25.42	6.02	19.40
	[POPS] (mol%)	Head	Tail	d_{PmB} along Z
Low	2	21.26	5.66	15.60
	5	18.93	5.53	13.40
	20	15.26	5.06	10.20
High	2	18.47	0	18.47
	5	17.07	0	17.07
	20	14.92	0	14.92

TABLE Supplementary Table 1. Output fitted positions of peak integrated density for head and tail components of PmB

System	n_{POPC}	n_{POPS}	n_{DMPS}	n_{PmB}	Simulation Run Time	$N_{Replicates}$
1	128	0	0	0	250	3
2	128	0	0	2	300	2
3	122	6	0	0	100	2
4	122	6	0	2	100	2
5	116	12	0	0	100	2
6	116	12	0	2	100	2
7	103	25	0	0	100	2
8	103	25	0	2	300	2
9	122	0	6	0	100	2
10	122	0	6	2	100	3
11	116	0	12	0	100	2
12	116	0	12	2	100	3
13	103	0	25	0	100	2
14	103	0	25	2	300	2
15*	126	0	0	2	100	1
16*	124	2	0	2	100	1
17*	120	6	0	2	100	1
18*	100	26	0	2	100	1
19*	124	0	2	2	100	1
20*	120	0	6	2	100	1
21*	100	0	26	2	100	1
22†	128	0	0	10†	100	1
23†	100	26	0	10†	200	1
24†	100	0	26	10†	200	1
25†	0	128	0	10†	200	1
26†	0	0	128	10†	200	1
27†	126	0	0	2*+10†	200	1
28†	0	126	0	2*+10†	200	1
29†	0	0	126	2*+10†	200	1

TABLE Supplementary Table 2. Details of membrane compositions and simulation lengths. (* PmB was started off inserted initially, † PmB was added every 20 ns using computational titration.)

Bacterial Strain	Lipid A to Lipid Ratio	Predicted Change in Pen. Depth (Å)	Fold Increase in Resistance	MIC (μ g/mL)	Fold Increase	Reference(s)
A. baumannii	60.00	1.46	9.72	0.25	16.00	[1, 2]
P. aeruginosa	29.00	0.70	8.83	1.00	4.00	[3, 4]
E. coli	75.00	1.82	10.15	0.13	32.00	[4]
Klebsiella spp.	18.00	0.44	8.52	1.40	2.86	[4, 5]
Salmonella spp.	39.00	0.95	9.12	0.50	8.00	[6]
Shigella spp.	57.00	1.38	9.63	0.25	16.00	[1, 7]
S. typhimurium	58.00	1.41	9.66	0.50	8.00	[8]
S. flexneri	60.00	1.46	9.72	0.25	16.00	[9]

TABLE Supplementary Table 3. MIC values are also obtained from [10] if not otherwise given in references.

[1] Joon-hui Chung, Abhayprasad Bhat, Chang-Jin Kim, Dongeun Yong, and Choong-Min Ryu. Combination therapy with polymyxin b and netropsin against clinical isolates of multidrug-resistant acinetobacter baumannii. *Scientific Reports*, 6:28168, 2016.

[2] Mohd Hafidz Mahamad Maifiah, Soon-Ee Cheah, Matthew D Johnson, Mei-Ling Han, John D Boyce, Visanu Thamlikitkul, Alan Forrest, Keith S Kaye, Paul Hertzog, Anthony W Purcell, et al. Global metabolic analyses identify key differences in metabolite levels between polymyxin-susceptible and polymyxin-resistant acinetobacter baumannii. *Scientific reports*, 6:22287, 2016.

[3] Alexandre P Zavascki, Jian Li, Roger L Nation, Silvana V Superti, Afonso L Barth, Larissa Lutz, Fabiano Ramos, Márcio M Boniatti, and Luciano Z Goldani. Stable polymyxin b susceptibility to pseudomonas aeruginosa and acinetobacter spp. despite persistent recovery of these organisms from respiratory secretions of patients with ventilator-associated pneumonia treated with this drug. *Journal of clinical microbiology*, 47(9):3064–3065, 2009.

[4] Raquel F Epand, Jake E Pollard, Jonathan O Wright, Paul B Savage, and Richard M Epand. Depolarization, bacterial membrane composition, and the antimicrobial action of ceragenins. *Antimicrobial agents and Chemotherapy*, 54(9):3708–3713, 2010.

[5] Flávia Bartolleti, Bruna Mara Silva Seco, Carla Capuzzo dos Santos, Carolina Bragança Felipe, Mara Elisa Borsato Lemo, Tatiane da Silva Alves, Lilian F Passadore, Marcelo J Mimica, Suely Carlos Ferreira Sampaio, Alexandre Prehn Zavascki, et al. Polymyxin b resistance in carbapenem-resistant klebsiella pneumoniae, sao paulo, brazil. *Emerging infectious diseases*, 22(10):1849, 2016.

[6] MJ Osborn, JE Gander, E Parisi, and J Carson. Mechanism of assembly of the outer membrane of salmonella typhimurium isolation and characterization of cytoplasmic and outer membrane. *Journal of Biological Chemistry*, 247(12):3962–3972, 1972.

[7] Henrik Köhler, Sonia P Rodrigues, and Beth A McCormick. Shigella flexneri interactions with the basolateral membrane domain of polarized model intestinal epithelium: role of lipopolysaccharide in cell invasion and in activation of the mitogen-activated protein kinase erk. *Infection and immunity*, 70(3):1150–1158, 2002.

[8] Marina Devyatyarova-Johnson, Ian H Rees, Brian D Robertson, Malcolm W Turner, Nigel J

Klein, and Dominic L Jack. The lipopolysaccharide structures of salmonella enterica serovar typhimurium and neisseria gonorrhoeaedetermine the attachment of human mannose-binding lectin to intact organisms. *Infection and immunity*, 68(7):3894–3899, 2000.

[9] Rachael M Rossi, Lauren Yum, Hervé Agaisse, and Shelley M Payne. Cardiolipin synthesis and outer membrane localization are required for shigella flexneri virulence. *MBio*, 8(4):e01199–17, 2017.

[10] Shawn Vasoo. Susceptibility testing for the polymyxins: Two steps back, three steps forward? *Journal of clinical microbiology*, pages JCM–00888, 2017.
