# Supplementary material for: Membrane charge and lipid packing determine polymyxin-induced membrane damage
Source: Commun Biol. 2019 Feb 18;2:67. doi: 10.1038/s42003-019-0297-6 (PMC6379423; doi:10.1038/s42003-019-0297-6)
Supplement: Supplementary file 2 — Description of Additional Supplementary Files [file 42003_2019_297_MOESM2_ESM.docx]

**Description of Additional Supplementary Files**

**File Name**: Supplementary Data 1

**Description**: Supplementary Data 1 (excel file). Cyclic voltammograms of POPS- and DMPS-enriched membranes with imbedded PmB over time, and average calculated charge transferred from replicate CV curves at ten minute point.

**File Name**: Supplementary Data 2

**Description**: Supplementary Data 2 (excel file). Cyclic voltammograms of POPS- and DMPS-enriched membranes with imbedded PmB at 10 minute time point in triplicate with controls. These curve were used to calculate the average charge transferred per sample in Supplementary Data 1.

**File Name**: Supplementary Data 3

**Description**: Supplementary Data 3 (excel file). Averaged measured fluorescence (normalized and raw data) values from ANTS/DPX vesicle leakage studies for POPC, POPC/POPS, and POPC/DMPS over time with replicates.
